# Learning-Based Rate Control for High Efficiency Video Coding

**DOI:** 10.3390/s23073607

**Published:** 2023-03-30

**Authors:** Sovann Chen, Supavadee Aramvith, Yoshikazu Miyanaga

**Affiliations:** 1 Department of Electrical Engineering, Faculty of Engineering, Chulalongkorn University, Bangkok 10330, Thailand; 2 Multimedia Data Analytics and Processing Research Unit, Department of Electrical Engineering, Faculty of Engineering, Chulalongkorn University, Bangkok 10330, Thailand; 3 Chitose Institute of Science and Technology, Chitose 066-8655, Japan

**Keywords:** HEVC, learning-based rate control, PSO

## Abstract

High efficiency video coding (HEVC) has dramatically enhanced coding efficiency compared to the previous video coding standard, H.264/AVC. However, the existing rate control updates its parameters according to a fixed initialization, which can cause errors in the prediction of bit allocation to each coding tree unit (CTU) in frames. This paper proposes a learning-based mapping method between rate control parameters and video contents to achieve an accurate target bit rate and good video quality. The proposed framework contains two main structural codings, including spatial and temporal coding. We initiate an effective learning-based particle swarm optimization for spatial and temporal coding to determine the optimal parameters at the CTU level. For temporal coding at the picture level, we introduce semantic residual information into the parameter updating process to regulate the bit correctly on the actual picture. Experimental results indicate that the proposed algorithm is effective for HEVC and outperforms the state-of-the-art rate control in the HEVC reference software (HM-16.10) by 0.19 dB on average and up to 0.41 dB for low-delay P coding structure.

## 1. Introduction

Multimedia technology has been upgraded from one generation to another to fulfill daily needs such as television, telephones, computers, robots, etc. Numerous multimedia applications have been utilized, including digital versatile disc (DVD), digital television (TV) broadcasting, video telephony, video teleconferencing, video games, and other forms of video-on-demand. According to [1], the resolution of television broadcasting has been upgraded from standard-definition television (SDTV) to 8K ultra high definition (UHD), which requires a very high bit rate to transmit or store. Furthermore, the video demand on internet traffic is increasing, based on a statistical report in the “Cisco Annual Internet Report (2018–2023)”, a Cisco White Paper in 2018 in [2]. Thus, it strongly needs an effective video coding technique to reduce the network traffic load with good visual quality and a lower bit rate.

In general, video properties have four redundancy criteria: spatial redundancy, temporal redundancy, perceptual redundancy, and statistical redundancy, which can be eliminated by the video coding standard [3]. High efficiency video coding (HEVC) [4], an advanced video coding standard released in 2013 by ITU-T and ISO/IEC, can effectively remove the digital video redundancies and achieve a bit rate saving of about fifty percent at the same visual quality by comparing with the previous standard (H.264/AVC [3,5,6]). HEVC is built following the structure of the successful block-based hybrid video coding approach [7], the same as the H.264/AVC video coding standard. In addition, several advanced techniques are applied in HEVC to get efficient compressions, such as flexible partitioning using quad-tree structure, prediction modes [8], sample adaptive offset (SAO) [9], and the cutting-edge interpolation technique [10].

Moreover, HEVC needs to have a functional encoder control, known as rate control, to determine the optimum codec parameters to accomplish minimal rate–distortion (*R*–*D*) score [11]. Many codec parameters include modes selection, quad-tree structure, motion estimation, and quantization parameter (QP). In common, the rate control algorithms [11,12] are used to define the bit allocation and QP by fixing the other parameters to accomplish the target bit with consistent visual quality. Specifically, rate control needs to manipulate the number of bits from a constant bit rate (CBR) into each coding level, including the group of picture (GOP) level, picture level, and basic units known as macroblocks (MBs) in H.264/AVC. The QP is then regulated to achieve the pre-allocated bits for each coding level, where the larger number of QP leads to a smaller number of allocated bits and vice versa. Encoder controls typically implement a uniform bit allocation in a GOP structure and initialize the fixed encoding parameters for any video contents to preserve a short-term constant output bit rate in the CBR channel. As a result, this implementation faces an infeasible problem of accurately adjusting encoding parameters for each GOP frame. Accordingly, if the target bit is less than the output bits, the encoded bits will rack up in the encoder buffer, causing a buffer overflow. The target bit is greater than the output bits, which implies the buffer underflow. Hence, controlling the relationship between bit rate and QP is essential for maintaining picture quality throughout the video sequence, as buffer overflows and underflows have an undesired effect on video quality fluctuations. *Q*-domain rate control is a direct estimation that attempts to model a correlation function between bit rate and quantization; the bit allocation can be computed from the QP to allocate for residual information but not for non-residual information. This model can work well when the coding parameters are not very flexible. Another rate control algorithm called ρ-domain rate control is developed [12,13] by introducing a linear function that outputs the coding bit rate from the percentage of zeros among the quantized transform coefficient. The model is effective only if the size of the transform is fixed. Both *Q*-domain and ρ-domain rate controls are designed to assume a high correlation between bit rate and quantization. This assumption is not valid for the current video codec because the codec becomes progressively variable [4]. Thus, a robust rate control [11], named *R*–λ rate control, has been released to achieve the best balance between bit rate and distortion. This rate control attempts to improve the coding efficiency and rate control accuracy by using the Lagrangian method, λ, for rate–distortion optimization (RDO).

Although the aim of *R*–λ rate control is for HEVC to enhance the coding efficiency compared with the conventional methods, two difficulties still need to be solved in HEVC reference software [14], including inaccurate bit allocation and inaccurate λ estimation. For the bit allocation part, the bit consumption of each CU of the first picture is computed by applying one to all initial encoder parameters at the basic unit level. In other words, all CUs are encoded using the same rate control parameters as the picture level. In such a case, the rate control will cause a bit consumption imbalance in the CU due to the spatial characteristic of each CU and result in the error bits’ distribution affecting the overall quality control. In addition, the inaccurate bit consumption at each coding level affects the λ adjustment to accomplish the frame bit budget because λ and the bit allocation are highly correlated. Specifically, according to the previous encoding results and the statistical characteristics of the input source data, the encoder parameters are empirically inaccurate, resulting from performance degradation at scene changes.

Based on these considerations, we propose a learning-based mapping method between *R*–λ parameters and video content to achieve accurate target bit rates and preserve good video quality. We use a feedback re-encoding method for the intra-picture and inter-picture to distribute *R*–λ parameters adaptively related to picture pattern changes. Additionally, the convolutional neural network (CNN) model [15] is used to capture the powerful spatial representation of the local coding tree units (CTUs). This CNN model is trained on the ImageNet dataset [16]. By incorporating the CNN model with the *R*–λ rate control algorithm, we can accurately obtain the expected number of bits per CTU. Our problem is a constrained optimization problem, where, by obtaining the optimal encoder control parameters to minimize the distortion subject to a constraint, the actual bit rate consumption is less than the target bit rate. To solve the constrained optimization problem, there are two optimization methods, namely the gradient-based method [17,18] and the non-gradient-based method (known as the evolutionary algorithm) [19,20,21,22,23,24]. The gradient-based method is effective only when the constraints and objective or penalty function can be derived. Since our model aims to map the high-dimensional feature space of the CTU to the *R*–λ parameter with the goal of *R*–*D* optimization, which cannot directly derive the gradient information from the penalty function, the evolutionary algorithm (EA) is chosen to optimize the parameters of our model. There are several EAs such as evolution strategies (ES) [19], simulated annealing (SA) [20], genetic algorithm (GA) [21], and particle swarm optimization (PSO) [22]. Due to the simplicity and convergence speed characteristics of all EAs [24], PSO is the most powerful one and has been successfully implemented to solve various constrained optimization problems [25,26,27,28]. Comprehensively, PSO takes the value of the objective function and uses primitive mathematical operators to solve the social behavior of model parameters. Therefore, PSO is implemented in our model to find the best solution for mapping the characteristics of CTU and rate control parameters. Furthermore, we feed the semantic residue information to adjust the current parameters of rate control updating cross-picture. The main contributions of this paper can be summarized in three aspects:

(i) We propose a learning-based neural network to define the mapping between video contents and rate control parameters to assign CTU budgets correctly;
(ii) We introduce a particle swarm optimization algorithm to finalize the optimal parameters at the basic unit level to maintain the bit budget and obtain good visual video quality;
(iii) We enhance the rate control parameter updating by considering the semantic residue information of the actual inter-picture into rate control.

The rest of the paper is organized as follows. In the next section, we briefly summarize related work. Then, the learning-based parameters of *R*–λ are described. After that, the experimental results are given. Finally, concluding remarks are provided.

## 2. Related Works

In this section, we briefly review the existing rate control models: *R*–*Q* model, ρ-domain-based Rate-GOP, *R*–λ models, and deep learning based rate control.

### 2.1. *R*–*Q* Model

The *R*–*Q* model [29] has extended to HEVC encoder control, known as a pixel-wise unified *R*–*Q* model (URQ); the quadratic *R*–*Q* model is defined as in (1),

(1) $$R=aQ^{-1}+bQ^{-2}$$

where *R* presents as the target bit rate, *Q* is the quantization parameter, and *a* and *b* are the parameters related to the video characteristic. The bit allocation of the URQ model is proposed similarly to the rate control model in H.264/AVC, where the target bit is computed based on the mean absolute difference (MAD) corresponding to the quantization step. As a result, compared with the earlier HEVC video coding standard (HM6.0) [14], the visual quality of the URQ model is slightly improved. However, some issues have been discussed regarding *Q*-domain rate control [30,31], such that QP is an integer data type that may not be adjusted accurately to achieve a bit budget.

### 2.2. ρ-Domain-Based Rate-GOP

The enhanced *R*–*Q* model known as ρ-domain-based Rate-GOP is proposed in [32] by presenting a new relationship one-to-one quantized transform coefficient with target bit rate. It is formulated as in (2):

(2) $$R_{i}=\theta_{i}\left(1-\rho_{i}\right)$$

where $\theta_{i}$ and $\rho_{i}$ denote a parameter related to the video pattern and the percentage of zero transform coefficients of frame *i*, respectively. Additionally, the mapping between non-zero transform coefficients and QP is determined following the quadratic function to properly allocate the bit to non-zero transform units. Consequently, the ρ-domain-based Rate-GOP can significantly achieve better video quality than the *Q*-domain rate control. Although this indirect relationship between *R* and *Q* technique is advantageous, it is still difficult to adapt its estimation to the variable block size transform in HEVC.

### 2.3. *R*–λ Model

To overcome the limitations of the *R*–*Q* model mentioned above, a new type of encoder control with the hierarchical bit allocation for every picture in a GOP is proposed in [11], called *R*–λ rate control. First, the author proposed a hyperbolic function as a model to express the characteristics of the *R*–*D* relationship, as in (3):

(3) $$D\left(R\right)=C\cdot R^{-K}$$

where *C* and *K* are parameters related to video content. Then, to minimize (3), λ is determined as the slope of the model in (4).

(4) $$\lambda =-\frac{\partial D}{\partial R}=C\cdot K\cdot R^{-K-1}$$

(5) $$\Leftrightarrow \lambda =\alpha \cdot R^{\beta }\equiv \gamma \cdot D^{\tau }.$$

Therefore, λ can indicate the trade-off between bit rate and distortion. If λ is large, the lower bit rate will cause higher distortion. On the other hand, small λ results in a higher bit rate with lower distortion. In addition, a hierarchical bit allocation method [33] is used to allocate different picture weights corresponding to each picture position in the GOP to improve coding efficiency. Furthermore, the QP can be computed by giving λ for each coding level as in (6).

(6) QP=4.2005·ln(λ)+13.7122.

The rate control can obtain stable buffer occupation and codec improvements through the hierarchical bit allocation method and the novel relationship between λ and *R*. As a result, *R*–λ rate control is generally used in the advanced video coding standard. However, the *R*–λ model mainly considers the bit rate by ignoring the characteristics of the video content. Furthermore, the model initializes its parameters by sharing the same fixed constant from the frame to all CTU levels. These aspects can cause video quality degradation.

A distortion-based Lagrange multiplier is proposed in [34] to enhance the compressed video quality in HEVC. The authors used the equivalent of distortion *D* and λ instead of *R*–λ. Two main objective functions control the λ adjustment: mean square error (MSE) and absolute error. MSE is calculated from the original and reconstructed video content, while the absolute error is computed by subtracting between the actual and target bit budget. This technique is designed for the non-hierarchical structure of rate control. It can enhance the video quality by an average of 0.23 dB in the low-delay P configuration compared with non-hierarchical *R*–λ rate control. The *R*–λ model with a hierarchical structure achieves a higher video quality of 0.26 dB than the *R*–λ model without a hierarchical structure [11]. This ability of the hierarchical structure in *R*–λ makes it a common approach as the default HEVC general test condition in [35]. A video quality enhancement of the compressed video worked on *R*–λ with a hierarchical structure is proposed in [36]. The authors introduced a simple rate control parameter-sharing in a GOP structure (PS-GOP), achieving a higher video quality of 0.07 dB on average and up to 0.17 dB compared to the default HEVC reference software (HM-16.10) [14].

An inter-block dependency-based CTU-level rate control for HEVC is established in [37], known as the RCA model. This proposed RCA is inspired by the temporal-dependent RDO, which is formulated as the fusion between inter-block dependency and R–D characteristics. This proposed model has achieved a considerable PNSR enhancement. However, the spatial coding units have not been taken into consideration, which would result in parameter propagation errors at the early stage.

### 2.4. Deep Learning-Based Rate Control

A deep reinforcement learning-based rate control for the dynamic video sequences is designed in [38] to capture the experience gained from the various factors, including brightness, variance, and gradient of each coding unit during the coding process. The proposed model is structured following the Markov decision process in a continuous discrete space to obtain better PSNR and lower-quality fluctuation. Nevertheless, the reinforcement approach has limitations, including a high number of interactions required to learn an optimal policy and difficulty generalizing to new, unseen environments.

Under a random access configuration, a deep convolution features-driven rate control for the HEVC encoders is proposed [39]. The method involves utilizing a pre-trained VGG-16 model to extract perceptual features, which addresses the problem of the rate control estimation. However, the model has not generalized the visual characteristic mapping to the rate control parameter.

Hence, we propose effective *R*–λ parameters associated with the video content to improve the compressed video quality and maintain the bit budgets at the encoder side. The following section presents the proposed framework in detail.

## 3. Learning-Based Rate Control

**Figure 1:**
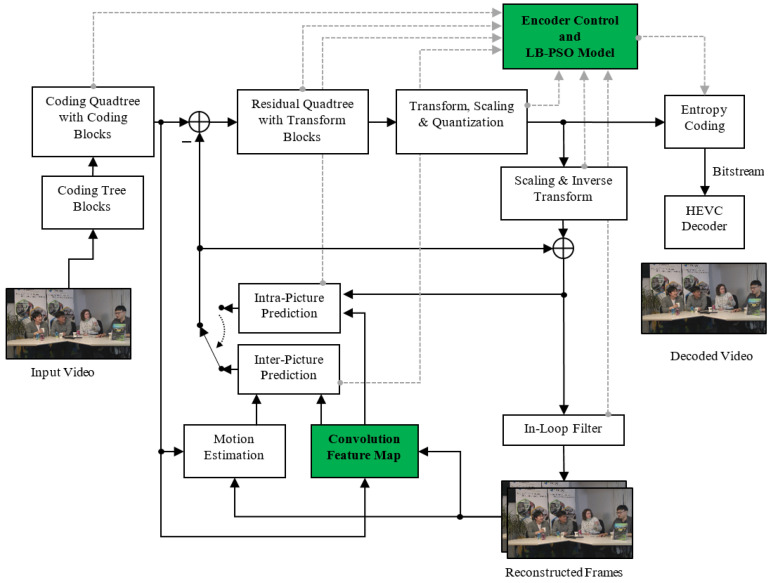
Learning-Based Rate Control Diagram for High Efficiency Video Coding.

**Figure 2:**
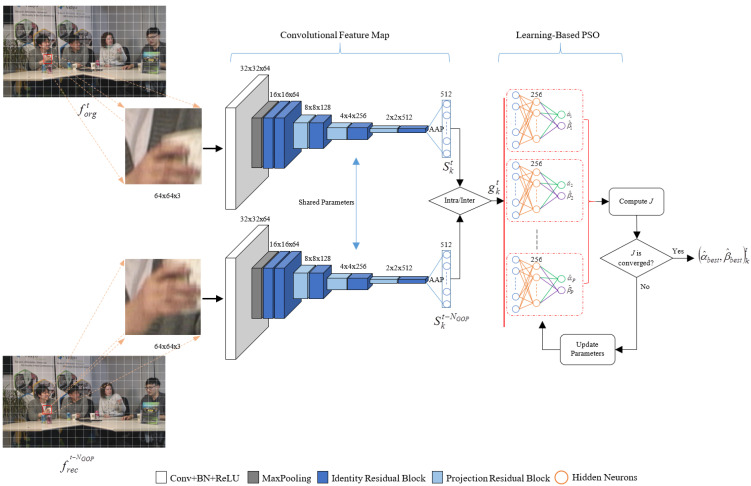
Overview of proposed learning-based particle swarm optimization.

This section introduces a learning-based rate control algorithm, which creates a regression map for the *R*–λ parameter. The proposed framework is designed, as shown in Figure 1. The green boxes represent the modification rate control model using the feature translation technique and the convolution feature map. First, the input video is fed into the convolution feature map to extract the high dimensional feature space, which contains essential features representing the CTU in the scene. Then, the proposed model learns to translate the input feature space to rate control parameters to get the optimal trade between the target bit rate and distortion rate. Additionally, the dashed lines from the inter- and intra-prediction are indicated to send the convolution feature representation of the video coding with the coding mode, whether intra- or inter-prediction to the Encoder Control block. Figure 2 shows the convolution feature map module and the regression map representations module, which are constructed to generate the *R*–λ parameters. The regression map is designed as learning-based particle swarm optimization (LB-PSO). Furthermore, the parameter updating for inter-coding is performed by considering residue information. The details of each part are presented in the following subsections.

### 3.1. Convolutional Feature Map

The convolutional feature map (fully convolutional networks—FCNs) is introduced at the first stage to obtain the meaningful spatial representation of CTU pictures for the input of our LB-PSO model. In general, the early layers of convolutions in the deep convolutional networks demonstrate the input image’s local or low-level feature information. In contrast, the deeper layers of convolutions indicate the high-level feature information that provides more global information about the image [40]. Additionally, the last fully connected (FC) layer of deep nets is designed to define the high-level feature information into object classes. Since FCNs do not include the FC layer, a relationship between the input image and the final feature output layer is preserved and expressed as data compression, which encodes the raw-pixel representation of the input image to high-level information. This information provides the global feature *G* representing the input image characteristic. *G* is fed into our LB-PSO model to generate the *R*–λ parameters. A pre-trained residual networks (ResNets) [15] model without the FC layer is used to extract the powerful convolutional feature. However, the original input size of ResNets is incompatible with the maximum size of CTUs. The adaptive average pooling (AAP) is then applied to the last convolution layers to ensure the compatibility of input and output dimensions. Figure 2 demonstrates the overall layout of our convolutional feature map architecture.

Suppose a *t*th frame contains a total *K* CTUs, then $G^{t}={\left\{g_{0},g_{1},\cdots ,g_{K}\right\}}_{t}$. Precisely, *G* is a parameter representing the high-dimensional features required as input to the proposed LB-PSO model. To obtain *G* for re-feedback coding of each coding structure in HEVC, i.e., intra- or inter-pictures, we define *G* as in (7):

(7) $$g_{k}^{t}=\left\{\begin{array}{cc} S_{k}^{t}, & \mathrm{if}\;\mathrm{intra-picture}.\\ {\left|S_{k}^{t}-S_{k}^{t-N_{GOP}}\right|}_{\left(t\;\mathrm{mod}\;c\right)}, & \mathrm{otherwise}. \end{array}\right.$$

where k∈K, and *c* (c>0) is a constant to determine the frame index for re-feedback coding on (tmodc). $N_{GOP}$ is the total number of pictures in a GOP. $S_{k}^{t}$ and $S_{k}^{t-N_{GOP}}$ represent the convolutional feature information (spatial representation) of $k^{th}$ CTU obtained from the original frame $f_{org}$ at *t* position and reconstruction frame $f_{rec}$ at $t-N_{GOP}$ position, respectively.

Specifically, if the encoding mode is intra coding, the spatial representation will directly input to the LB-PSO model. Otherwise, we compute the semantic residue information by applying the absolute difference between the current spatial representation $S_{k}^{t}$ of the original CTU and the previous spatial representation $S_{k}^{t-N_{GOP}}$ of the reconstructed CTU before feeding it to the LB-PSO model to accurately generate rate control parameters on the changes between consecutive CTUs. In addition, the reconstructed frame at $t-N_{GOP}$ is chosen in the proposed method because a group of pictures in a video allows for exploits of the temporal redundancy in the video. The proposed model can be adapted following the $N_{GOP}$.

### 3.2. Learning-Based Particle Swarm Optimization Network

#### 3.2.1. LB-PSO Estimator

Our LB-PSO is proposed to define the optimal mapping ϕ from the spatial–temporal representation of CTU $g_{k}$ to rate control parameters $y_{k}$, $y_{k}={\left\{\alpha ,\beta \right\}}_{k}$. We introduce a feedforward network with one hidden layer to determine $y_{k}$. This feedforward network can be computed as in (8):

(8) $$y_{k}=\phi \left(h_{k};W_{\phi },b_{\phi }\right)=W_{\phi }^{T}h_{k}+b_{\phi }$$

where $W_{\phi }$ provides the weights of a mapping function ϕ, $b_{\phi }$ is a bias, and $h_{k}$ represents the output of the hidden layer. Precisely, $h_{k}$ is designed by applying a rectified linear activation function to the output of a linear transformation composed of the weights $W_{h}$ and bias $b_{h}$ parameters to trigger a non-linear transformation. Thus, $h_{k}$ can be derived as in (9):

(9) $$h_{k}=\max\left\{0,W_{h}^{T}g_{k}+b_{h}\right\}$$

From (8) and (9), our complete mapping model can be reformulated as in (10):

(10) $$y_{k}=W_{\phi }^{T}\max\left\{0,W_{h}^{T}g_{k}+b_{h}\right\}+b_{\phi }$$

The model parameters $M=\{W_{\phi },W_{h},b_{\phi },b_{h}\}$ are optimized by utilizing swarm intelligence to exchange information between particles about *R*–*D* cost function, *J*. On the other hand, the model parameters regulate its trajectory concerning its best previous position and the best previous position reached by any member of its neighborhood. To target the swarm intelligence rule, the cost function *J* is determined by two objective functions, including a reconstruction error (MSE) of visual quality and smooth ${}_{L1}$ error of bit allocation. The cost function *J* can be defined as in (11):

(11) $$J=\frac{1}{N}\sum_{j=0}^{N-1}{\left(f_{org_{j}}-f_{rec_{j}}\right)}^{2}+\eta {\mathrm{smooth}}_{L1}\left(R_{T}-R_{A}\right)$$

(12) $${\mathrm{smooth}}_{L1}\left(U\right)=\left\{\begin{array}{cc} \frac{U^{2}}{2}, & \mathrm{if}\;\left|U\right|<1\\ \left|U\right|-\frac{1}{2}, & \mathrm{otherwise} \end{array}\right.$$

where *N* is the total number of pixels in a picture and η is a penalty coefficient. $R_{T}$ and $R_{A}$ are the target and actual bit on the picture level, respectively.

According to the cost function design, the model parameters are updated after all CTUs are fully encoded. This cost function is aimed at the model learning to achieve the trade-off between distortion and bit allocation. The next section introduces the complete process of the parameters update.

#### 3.2.2. Parameter Updating

In this subsection, we present the parameter update of the encoder controller corresponding to the intra/inter coding mode. In addition, the inter coding mode is classified into two sets of coding frames: a core frame and a common one. A core frame is encoded by activating the re-feedback coding to adjust the bit budget at the CTU coding level. In contrast, the common frame is coded by applying the default Lagrangian multiplier to determine the bit budget at the CTU coding level. For both intra coding and core frame of inter coding, the bit budget at the CTU coding level is computed using Equations (4) and (10). Additionally, the model parameters *M* in Equation (10) individually parameterize its value according to its movement in a search space.

Let *P* be the total size of the population, $V_{i}$ be the velocity (position change) of *i*-th particle, $B_{i}$ be the best previous model parameters of *i*-th particle, and $B_{g}$ be the best model parameter in the swarm. Then the swarm is manipulated on each iteration *n* according to the following two equations:

(13) $$V_{i}^{n+1}=aV_{i}^{n}+c_{1}r_{i1}^{n}\left(B_{i}^{n}-M_{i}^{n}\right)+c_{2}r_{i2}^{n}\left(B_{g}^{n}-M_{i}^{n}\right),$$

(14) $$M_{i}^{n+1}=M_{i}^{n}+V_{i}^{n+1},$$

where i=1,2,⋯,P, and *a* is the inertia weight of velocity *V*, which is used to control the trade-off between the swarm’s global and local exploration capabilities. $c_{1}$ and $c_{2}$ are two positive acceleration constants, named the PSO’s cognitive and social parameters, respectively. $r_{i1}$ and $r_{i2}$ are the random numbers, generated from a uniform distribution within the range [0,1]. The performance of each model parameter $M_{i}$ in the swarm is measured according to the cost function *J*. The lower cost function indicates a better $M_{i}$. After finalizing the best $M_{i}$ to preserve the minimal cost function *J* at the CTU coding level, the CTU is encoded.

For the picture level of inter coding, the rate control parameters are adjusted by considering the residue score of the semantic residue information. The probability of residue score $Q^{t}$ on a picture at time *t* can be computed as

(15) $$Q^{t}=\sum_{k\in K}\sum_{j\in S_{k}}\frac{A_{k}^{t}\left(j\right)}{S_{k}^{t}\left(j\right)}$$

(16) $$A_{k}^{t}\left(j\right)=\left\{\begin{array}{cc} 0, & \mathrm{if}\;t-N_{GOP}\leq 0\\ \left|S_{k}^{t}\left(j\right)-S_{k}^{t\times \left\lfloor \frac{t}{N_{GOP}}\right\rfloor }\left(j\right)\right|, & \mathrm{otherwise} \end{array}\right.$$

where $\left\lfloor .\right\rfloor$ represents the rounded result. Additionally, in the GOP regarding the spatiotemporal information of the video sequence, the picture levels generally have different pairs of encoder controller coefficients $\alpha_{p}$ and $\beta_{p}$. Therefore, the rate control parameters can be updated by (17)–(21). The Lagrangian multiplier, λ, is defined as

(17) $$\lambda =\alpha_{pold}\cdot R^{\beta_{pold}}$$

If the $GOP_{id}$ equals 0, a pair of rate control parameters can be formulated as in (18) and (20).

(18) $$\alpha_{pnew}=\alpha_{pold}+\delta_{\alpha }\cdot \left(\ln\left(\lambda_{r}-\lambda_{c}\right)\right)\cdot \alpha_{pold}+\zeta Q^{t}$$

(19) $$\beta_{pnew}=\beta_{pold}+\delta_{\beta }\cdot \left(\ln\left(\lambda_{r}-\lambda_{c}\right)\right)\cdot \ln\left(bpp_{r}\right)+\frac{\zeta }{2}Q^{t}$$

Otherwise, a pair of rate control parameters can be computed as in (19) and (20).

(20) $$\alpha_{pnew}=\alpha_{pold}+\zeta Q^{t}$$

(21) $$\beta_{pnew}=\beta_{pold}+\frac{\zeta }{2}Q^{t}$$

where $\delta_{\alpha }$ and $\delta_{\beta }$ are the default constant in HEVC reference software. $\lambda_{r}$ represents the real λ value, $\lambda_{c}$ is a computed λ value from the real cost $bpp_{r}$ with the previous rate control parameters $\alpha_{pold}$ and $\beta_{pold}$ at picture level, and ζ is the residue penalty constant.

For the quantization parameter (QP), it can be determined as in (21).

(22) QP=4.2005·ln(λ)+13.7122

**Figure 3:**
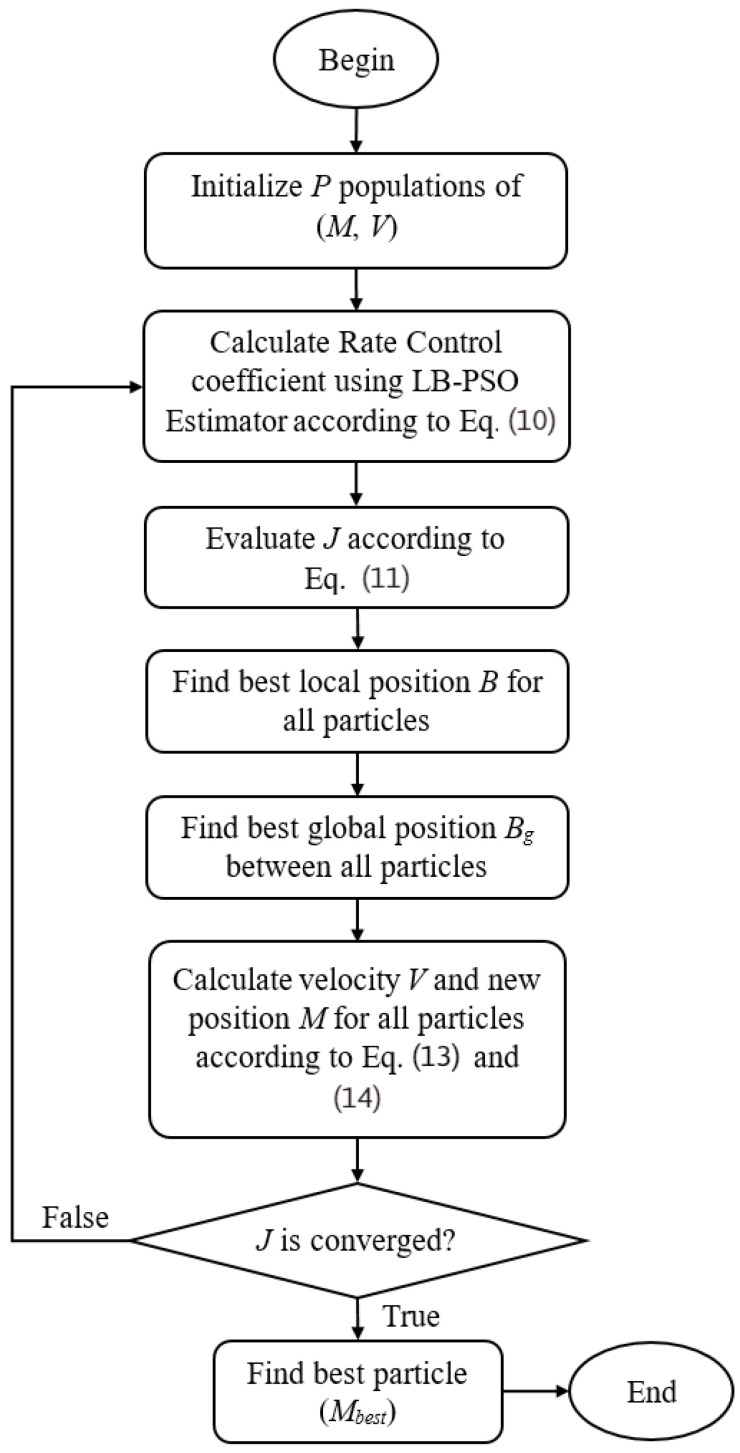
Learning-based particle swarm optimization flowchart.

Figure 3 provides the model flowchart of the learning-based PSO method, named LB-PSO. LB-PSO initially randomizes the group of particle parameters. Then, the rate control coefficients are computed using the LB-PSO estimator. Subsequently, the LB-PSO model’s best local and global parameters have reallocated if the current position is better than the stored position according to its cost function, *J*. After that, the velocity *V* and position *M* are calculated following Equations (13) and (14). Finally, the best particle for the LB-PSO model is determined to generate the best rate control coefficients for the current input CTU context.

## 4. Experimental Results

To evaluate the performance of the proposed learning-based particle swarm optimization, the experiments are conducted on various videos, including static and dynamic scenes.

### 4.1. Experiment Setting

In the experiment, the proposed algorithm is implemented on HEVC reference software [14] and is compared with the PS-GOP [36] and the state-of-the-art *R*–λ rate control (RC-HEVC) [11]. According to HEVC common parameter setting [3], the largest size of a CTU produces high-efficiency coding performance. Specifically, the largest feasible size of a CTU in HEVC is a 64 × 64 block size. We have also designed the model to adapt bit allocation for CTUs related to their spatial information, which is extracted using a pre-trained CNN model. Since we have implied CNN feature extraction on the largest size of a CTU in HEVC, we transform YUV420 format to a true color (64 × 64 × 3) CTU as the input in the feature extraction block. The proposed algorithm and baseline methods are simulated in the same reference software HM-16.10. Precisely, the experiments are conducted under the low-delay P main profile configurations, and the encoder parameters are set according to the standard setting in [35] by enabling the rate control as True. In addition, there are 100 iterations in every decision-making process for each rate control parameters prediction in the proposed LB-PSO. There are fifteen test video sequences with four video resolutions, including two videos of 240p (wide quarter video graphics array—WQVGA) [41], three videos of 480p (wide video graphics array—WVGA) [41], five videos of 720p (HD) [42], three videos of 1080p (full HD) [41], and two videos of 4k resolution [43]. Table 1 briefly summarizes the characteristics of the test video sequence. In addition, the test video sequence is encoded at four target bit rates corresponding to the video resolution. Since the goal of rate control is not only to improve the visual quality of the video for a given bit rate but also to achieve the bit rate closest to the target bit rate, both peak signal-to-noise ratio (PSNR) and bit rate error (BRE) are used as the criteria for determining the performance of the rate control algorithm. The PSNR and BRE can be computed as in (22) and (23).

(23) $$PSNR=10\log\left[\frac{{\left(2^{n}-1\right)}^{2}}{\frac{1}{N}\sum_{j=0}^{N-1}{\left(f_{org_{j}}-f_{rec_{j}}\right)}^{2}}\right]$$

(24) $$BRE=\frac{\left(R_{T}-R_{A}\right)}{R_{T}}\times 100\%$$

where *n* represents bit depth.

### 4.2. Experimental Results and Analysis

*(1) R–D performance and Bit Rate Accuracy*: The first experiment was conducted on the low video resolution (WQVGA), which contains two video sequences with different frame rates, including BlowingBubbles and BQSquare. These two videos have various dynamic characteristics, such as a moving camera, moving objects, and illumination changes. Table 2 describes the proposed method’s PSNR and BRE performance compared with the baseline methods. Our learning-based method outperforms all the baseline methods as we achieve the highest PSNR value with the same bit rate.

**Figure 4:**
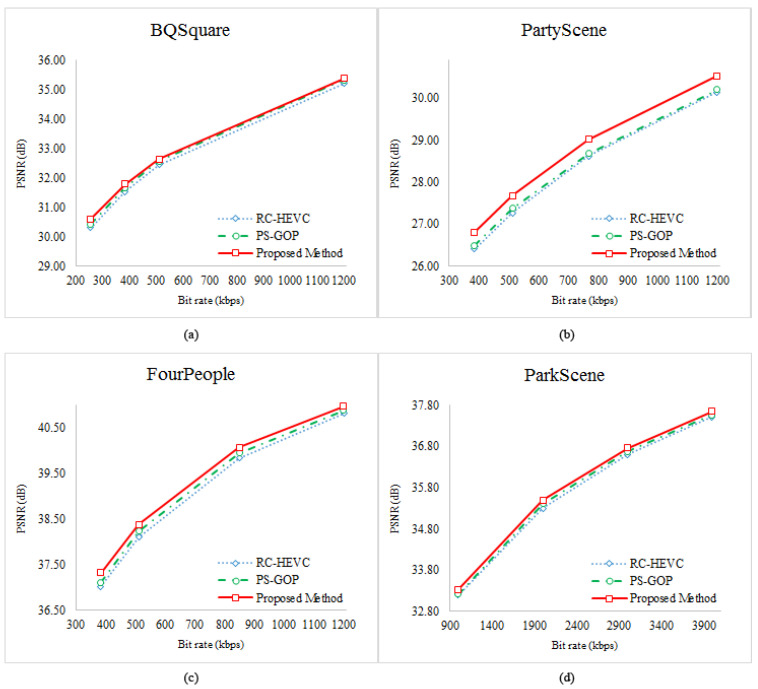
Rate–Distortion curves: (**a**) BQSquare, (**b**) PartyScene, (**c**) FourPeople, (**d**) ParkScene.

Specifically, our method’s average PSNR enhancement is 0.23 dB and 0.12 dB compared with RC-HEVC and PS-GOP, respectively. Our approach also performs the maximum PSNR improvement (max) of 0.30 dB and 0.20 dB compared to RC-HEVC and PS-GOP. Figure 4a illustrates the *R*–*D* performance curve of the BQSquare test sequence. The learning-based approach obtains a better *R*–*D* performance than the baselines method. In addition, the average BRE of RC-HEVC, PS-GOP, and our methods are 0.01%, indicating that all approaches can effectively achieve the target bit rate. However, the proposed method has the lowest BRE at a lower target bit rate (256 kbps). It is noticed that the RC-HEVC has poor visual quality on these WQVGA with dynamic scenes compared to all approaches. As a result, even if the scene has dynamic properties, our algorithm can constructively achieve the target bit rate with the good visual quality of the WQVGA sequence.

Next, the WVGA sequences were tested, such as BasketballDrillText, PartyScene, and BQMall. The scene properties are similar to the above experiments, but these WVGA sequences are more challenging than WQVGA because they involve multi-object movement, camera movement, and higher resolution. The outcomes of PSNR and BRE are summarized in Table 3, where the proposed learning-based method works much better. It reaches 0.41 dB and 0.33 dB of visual quality better than RC-HEVC and PS-GOP, respectively. Concisely, our approach has no error bit consumption on average and performs 0.23 dB and 0.16 dB on average higher than RC-HEVC and PS-GOP, respectively. On one side of the *R*–*D* curve, our proposed method is significantly higher than the competitive methods, as shown in Figure 4b. Based on the outcomes of all approaches in Table 2 and Table 3, the *R*–λ rate control and PS-GOP are unsuitable for such dynamic scenes and cameras. Consequently, it can indicate that the λ adjustment and quality control are not correctly estimated.

After testing the WVGA sequences, the HD videos containing video conferencing and online teaching test sequences were simulated. The HD videos are FourPeople, KristenAndSara, Vidyo1, Vidyo3, and Vidyo4. These videos have the characteristics of a static camera with multiple objects moving. Figure 4c shows an overall outgrowth of the *R*–*D* curve of FourPeople from the low bit rate to the high bit rate. Although the scene is used with a static camera, the proposed method’s *R*–*D* performance is noticeably greater than the competitive methods. Additionally, the PSNR and BRE evaluations of these HD video sequences are recorded in Table 4, where the average PSNR enhancement value of our method is approximately 0.17 dB (max = 0.30 dB) and 0.08 dB (max = 0.21 dB) in comparison with the RC-HEVC and PS-GOP.

The last experiment was applied on full HD and 4k video test sequences. The first three videos, ParkScene, Cactus, and BQTerrace, were used for the full HD experiment. The last two sequences, HoneyBee and Jocky, were used for 4k videos. This last test contained all types of scenarios. The ParkScene and Jocky videos have a moving camera and multiple object motions, while the BQTerrace video stacks the camera motion with a static camera. Furthermore, the Cactus video consists of a static camera and the rotation of the objects. The HoneyBee video has multiple object motions and a static camera. According to Table 5, the overall PSNR evaluation of the proposed method on the BQTerrace sequence at a low bit rate is the highest compared to the other sequences. In contrast, the ParkScene sequence has the highest PSNR at a high bit rate. The reason is that the scenes containing a dynamic camera have significant movement changes; thus, the state-of-the-art *R*–λ rate control cannot update the encoding controller correctly. In addition, PS-GOP uses parameter sharing in GOP, which is not enough to adapt to encoder parameters following frame characteristics. Reasoning from this fact, our method establishes a novel mapping between frame features and *R*–λ coefficient parameters. We provide a computationally feasible solution using LB-PSO to produce optimal *R*–*D* for good visual quality and to maintain the target bit rate. Figure 4 shows the overall *R*–*D* curve on different video resolutions. Consequently, our method has achieved the highest outcomes of all competitive methods. From Table 2 to Table 5, the average PSNR improvement is 0.19 dB (max = 0.41 dB) and 0.10 dB (max = 0.33 dB) compared with RC-HEVC and PS-GOP, respectively.

The PSNR performance of our proposed model is extensively compared with other state-of-the-art rate control methods for both the dynamic scene and interview scene as shown in Table 6. Our proposed model achieves the highest PSNR for all bit rates in both types of video sequences. This indicates that the inter coding approach should not only consider the inter-block dependency coding structure but also the rate control coefficient.

**Figure 5:**
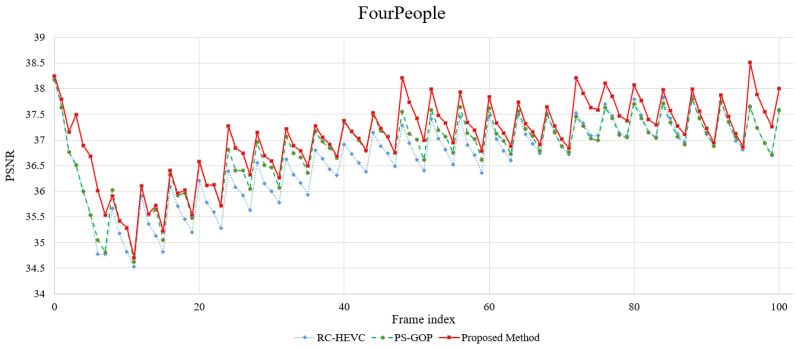
Comparison of PSNR difference between consecutive frames.

**Figure 6:**
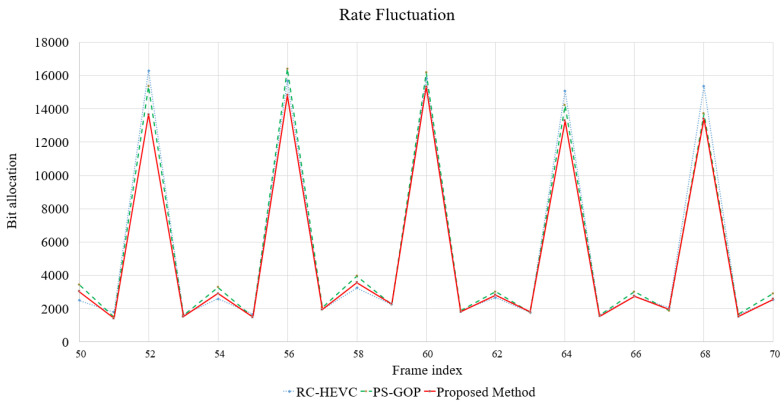
Rate fluctuation performance comparison.

Additionally, Figure 5 shows a graph of the PSNR difference between consecutive frames. The plot shows that the performance of the proposed method adaptively achieves better results on frame reconstruction from the start of encoding compared to RC-HEVC and PS-GOP. This demonstrates the effective interaction of spatiotemporal features in the rate control model and the crossed LB-PSO model to decide on appropriate rate control coefficients to acquire the target bit rate and perform well in PSNR. Furthermore, Figure 6 indicates the details of the rate fluctuation performance of the proposed method compared to the baselines. This rate fluctuation describes successive frames’ historical bit allocation performance to understand the bit flow in the video codec. Therefore, LB-PSO can control bit allocation better than the baselines, and it can carry out lower bit allocation and produce higher PSNR in most consecutive frames, as shown in Figure 5 and Figure 6.

**Figure 7:**
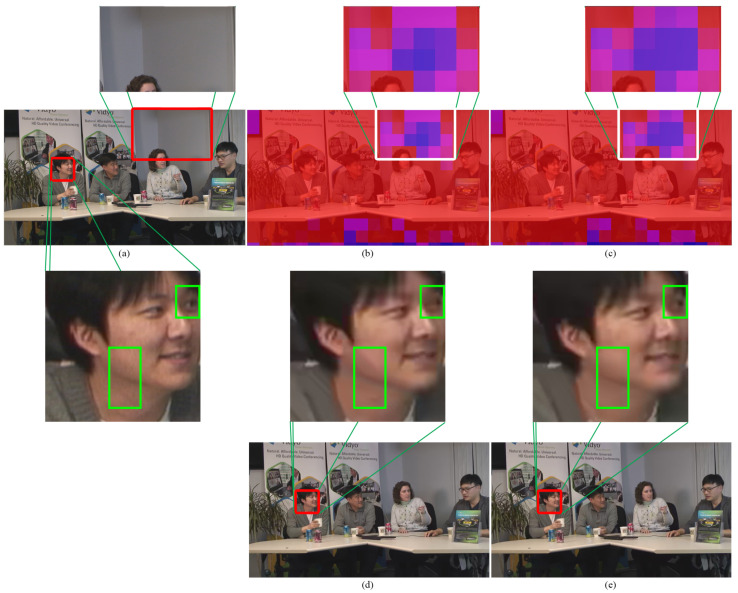
Bit heatmaps and reconstructed frame of intra coding at 384 kbps: (**a**) original frame, (**b**,**d**) RC-HEVC, and (**c**,**e**) proposed method.

**Figure 8:**
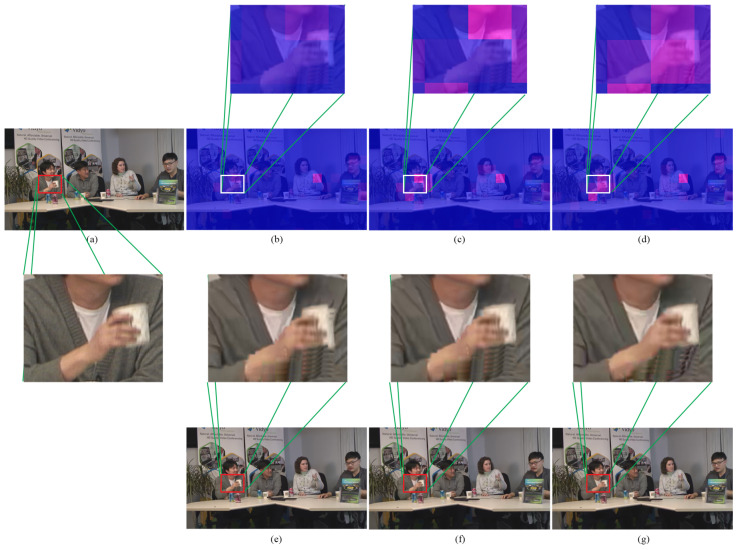
Bit heatmaps and reconstructed frame of inter coding at 384 kbps: (**a**) original frame, (**b**,**e**) RC-HEVC, (**c**,**f**) PS-GOP, and (**d**,**g**) proposed method.

*(2) Bit Heatmaps and Visual Quality*: To indicate the performance of bit allocation at the CTU level, the heatmap visualization and the subjective results of the reconstructed frame are illustrated in Figure 7 and Figure 8. Since there is no modification of the intra coding of PS-GOP, Figure 7 shows only the comparison between state-of-the-art RC-HEVC with our proposed learning-based approach. The bit consumption is highlighted by red color intensity on each CTU, while the blue acts as a mask to cover the frame. If the red intensity is low, the allocated bits are consumed less. The patch image is extracted from the frame to illustrate the greatest difference in bit consumption at the CTU level of RC-HEVC and our proposed method. Figure 7b,c reveal that the bit allocation performance of RC-HEVC on the plane space CTU is slightly high, leading to less bit budget for the necessary spatial CTU. On the contrary, our proposed method obtains smoother bit allocation on non-important spatial images (low-frequency components), providing more budget to important CTU features. Additionally, the visualization of the human face of the proposed learning-based approach on the intra-picture shows more details with a smoother look than that of RC-HEVC, as shown in the green box of Figure 7c,d. According to these results, our LB-PSO can obtain better bit allocation by using the information from the mapping encoder control parameters with the input convolution feature map of each spatial CTU instead of the fixed initialization of *R*–λ rate control.

For inter coding, the PS-GOP is added in comparison. Similarly, the color representation is defined the same as the intra coding. Regarding bitmaps, Figure 8b shows that RC-HEVC has a problem with bit allocation on the essential features. Due to hand movement, RC-HEVC should provide higher bit allocation in these necessary parts; on the contrary, it allocates fewer bits to these blocks. Furthermore, PS-GOP attempts to allocate the amount of bit budget to the hand movement area to keep the visual quality of the action consistent. However, the bit budget on large hand motion blocks is still small, as shown in Figure 8c.

Regarding residual semantic information, our proposed method can correctly regulate the bit budget responding to the motion information in the scene, as illustrated in Figure 8d. On the other hand, our proposed method obtains the accurate bit allocation of each CTU corresponding to its spatial–temporal characteristics. Furthermore, the visual quality visualization of this hand movement is shown in Figure 8e–g. In particular, RC-HEVC has a considerable distortion in this hand movement area, while PS-GOP is slightly better than RC-HEVC. Although PS-GOP is better than RC-HEVC, PS-GOP still has higher distortion compared with our proposed method. As a result, the proposed method achieves better hand and cup shapes than the competitive methods. According to our experimental results, we can conclude that the proposed learning-based *R*–λ parameter outperforms other competing methods by achieving the highest PSNR while maintaining the target bit rate.

*(3) Computational Complexity*: We compare the computational time of the proposed method with RC-HEVC and PS-GOP. Regarding computational time in an average of seconds per frame, as indicated in Table 7, our LB-PSO achieves 53.30 s/frame, 97.79 s/frame, and 351.10 s/frame on WVGA, HD, and full HD resolution, respectively. We also compare our computational complexity with other baseline methods. Table 6 shows that our computational time is higher than the baseline methods. This is because our framework is designed as online training using the integration of the forward pass network with particle swarm optimization. However, we obtained a significantly higher PSNR value and achieved the target bit rate. Furthermore, our bit allocation was assigned correctly compared to baseline approaches.

## 5. Conclusions

In this paper, we proposed novel learning-based *R*–λ parameters for HEVC. The proposed framework is embedded with a deep convolution neural network feature map and LB-PSO, which brings advantages to rate control parameters estimation corresponding to spatial–temporal CTUs. LB-PSO is designed to obtain the feasible rate control coefficient parameters solution to optimize the *R*–*D* relationship. Experimental results clearly show that our proposed learning-based approach obtains an accurate target bit rate with 0.19 dB on average to 0.41 dB and 0.10 dB on average to 0.33 dB maximum PSNR improvement than the state-of-the-art RC-HEVC and PS-GOP, accordingly. Due to the bit allocation, our algorithm can achieve an operational bit distribution to each CTU on both intra and inter coding. In other words, our method is effective and robust for determining the bit budget for the CTU of the frame. For future work, CTU partitioning will be considered together with *R*–λ parameters to increase coding efficiency.

## Figures and Tables

**Table 1:** Characteristics of Test Video Sequences and Bit Rate.

Resolution	Name of Video Sequence	Frame Rate (fps)	Bit Rate (kbps)
3840 × 2160	HoneyBee	120	1000, 2000, 3000, 4000
	Jocky	120	
1920 × 1080	ParkScene	24	1000, 2000, 3000, 4000
	Cactus	50	
	BQTerrace	60	
1280 × 720	FourPeople	60	384, 512, 850, 1200
	KristenAndSara	60	
	Vidyo1	60	
	Vidyo3	60	
	Vidyo4	60	
832 × 480	BasketballDrillText	50	384, 512, 768, 1200
	PartyScene	50	
	BQMall	60	
416 × 240	BlowingBubbles	50	256, 384, 512, 1200
	BQSquare		60

**Table 2:** The Performance of PSNR and BRE of Video Sequence with Resolution of 416 × 240.

Name of Video Sequence	Target Bit Rate	RC-HEVC			PS-GOP			Proposed Method		
		Bit Rate	PSNR	BRE	Bit Rate	PSNR	BRE	Bit Rate	PSNR	BRE
BlowingBubbles	256	256.06	29.69	−0.02	256.08	29.79	−0.03	256.02	29.99	−0.01
	384	384.05	31.14	−0.01	384.00	31.26	0.00	384.02	31.44	−0.01
	512	512.06	32.26	−0.01	512.05	32.38	−0.01	512.04	32.51	−0.01
	1200	1200.18	35.64	−0.02	1200.05	35.71	0.00	1200.15	35.73	−0.01
BQSquare	256	256.04	30.31	−0.02	256.01	30.42	−0.01	256.02	30.60	−0.01
	384	384.03	31.53	−0.01	384.03	31.67	−0.01	384.03	31.78	−0.01
	512	512.03	32.45	−0.01	512.03	32.56	−0.01	512.02	32.64	0.00
	1200	1200.06	35.20	0.00	1200.04	35.33	0.00	1200.04	35.37	0.00
Average			32.28	−0.01		32.39	−0.01		**32.51**	−0.01

**Table 3:** The Performance of PSNR and BRE of Video Sequence with Resolution of 832 × 480.

Name of Video Sequence	Target Bit Rate	RC-HEVC			PS-GOP			Proposed Method		
		Bit Rate	PSNR	BRE	Bit Rate	PSNR	BRE	Bit Rate	PSNR	BRE
BasketballDrillText	384	384.03	30.82	−0.01	383.99	30.93	0.00	384.02	30.99	−0.01
	512	512.05	31.94	−0.01	512.00	32.01	0.00	511.99	32.08	0.00
	768	768.04	33.46	−0.01	768.04	33.52	−0.01	768.05	33.60	−0.01
	1200	1200.10	35.15	−0.01	1200.07	35.20	−0.01	1200.07	35.32	−0.01
PartyScene	384	384.01	26.40	0.00	384.00	26.49	0.00	383.97	26.80	0.01
	512	512.02	27.27	0.00	512.01	27.37	0.00	511.96	27.68	0.01
	768	768.09	28.61	−0.01	768.02	28.68	0.00	768.02	29.01	0.00
	1200	1200.06	30.15	−0.01	1200.02	30.20	0.00	1200.03	30.53	0.00
BQMall	384	384.01	30.68	0.00	384.13	30.77	−0.03	384.00	30.85	0.00
	512	512.01	31.86	0.00	512.05	31.92	−0.01	512.03	32.00	−0.01
	768	768.01	33.50	0.00	768.01	33.59	0.00	768.01	33.66	0.00
	1200	1200.04	35.28	0.00	1200.03	35.33	0.00	1200.01	35.39	0.00
Average			31.26	−0.01		31.33	−0.01		**31.49**	0.00

**Table 4:** The Performance of PSNR and BRE of Video Sequence with Resolution of 1280 × 720.

Name of Video Sequence	Target Bit Rate	RC-HEVC			PS-GOP			Proposed Method		
		Bit Rate	PSNR	BRE	Bit Rate	PSNR	BRE	Bit Rate	PSNR	BRE
FourPeople	384	383.97	37.02	0.01	383.99	37.12	0.00	383.99	37.32	0.00
	512	511.97	38.10	0.01	512.00	38.24	0.00	511.99	38.38	0.00
	850	849.98	39.84	0.00	849.99	39.94	0.00	849.98	40.06	0.00
	1200	1200.08	40.81	−0.01	1199.96	40.87	0.00	1200.05	40.97	0.00
KristenAndSara	384	384.06	39.17	−0.02	384.08	39.32	−0.02	384.12	39.37	−0.03
	512	512.07	40.03	−0.01	512.09	40.17	−0.02	512.11	40.20	−0.02
	850	850.12	41.31	−0.01	850.09	41.43	−0.01	850.12	41.47	−0.01
	1200	1200.18	42.04	−0.01	1200.16	42.12	−0.01	1200.16	42.16	−0.01
Vidyo1	384	384.00	38.95	0.00	383.98	39.06	0.01	384.00	39.11	0.00
	512	512.01	39.86	0.00	511.93	39.95	0.01	511.99	40.01	0.00
	850	849.96	41.19	0.00	849.88	41.26	0.01	850.01	41.32	0.00
	1200	1200.00	41.93	0.00	1199.96	42.00	0.00	1200.01	42.07	0.00
Vidyo3	384	384.01	37.85	0.00	384.00	38.00	0.00	384.02	38.01	−0.01
	512	512.02	38.82	0.00	512.01	38.95	0.00	512.01	38.97	0.00
	850	850.01	40.22	0.00	850.01	40.33	0.00	850.01	40.37	0.00
	1200	1200.02	41.00	0.00	1200.03	41.08	0.00	1200.00	41.12	0.00
Vidyo4	384	384.01	38.68	0.00	384.01	38.73	0.00	384.01	38.86	0.00
	512	512.02	39.47	0.00	512.01	39.53	0.00	512.02	39.67	0.00
	850	850.02	40.67	0.00	850.01	40.74	0.00	850.02	40.86	0.00
	1200	1200.02	41.39	0.00	1200.05	41.45	0.00	1200.02	41.54	0.00
Average			39.92	0.00		40.02	0.00		**40.09**	0.00

**Table 5:** The Performance of PSNR and BRE of Video Sequence with Resolution of 1920 × 1080 and 4k.

Name of Video Sequence	Target Bit Rate	RC-HEVC			PS-GOP			Proposed Method		
		Bit Rate	PSNR	BRE	Bit Rate	PSNR	BRE	Bit Rate	PSNR	BRE
ParkScene	1000	999.96	33.20	0.00	999.84	33.21	0.02	999.86	33.32	0.01
	2000	2000.01	35.30	0.00	1999.89	35.41	0.01	2000.10	35.49	0.00
	3000	2999.95	36.60	0.00	2999.91	36.68	0.00	2999.98	36.76	0.00
	4000	4000.11	37.52	0.00	4000.09	37.57	0.00	4000.11	37.66	0.00
Cactus	1000	1000.01	31.62	0.00	1000.02	31.75	0.00	1000.02	31.74	0.00
	2000	2000.04	33.77	0.00	2000.03	33.85	0.00	2000.03	33.87	0.00
	3000	3000.09	34.96	0.00	3000.03	35.01	0.00	3000.03	35.04	0.00
	4000	4000.06	35.70	0.00	3999.95	35.77	0.00	4000.07	35.81	0.00
BQTerrace	1000	1000.05	31.62	−0.01	1000.01	31.73	0.00	1000.17	31.97	−0.02
	2000	2000.13	33.03	−0.01	2000.02	33.11	0.00	2000.04	33.25	0.00
	3000	3000.15	33.67	0.00	3000.01	33.78	0.00	3000.08	33.82	0.00
	4000	4000.53	34.10	−0.01	4000.05	34.20	0.00	4000.04	34.15	0.00
HoneyBee	1000	1000.01	38.24	0.00	1000.00	38.25	0.00	1000.03	38.31	0.00
	2000	2000.01	38.63	0.00	2000.00	38.65	0.00	2000.01	38.66	0.00
	3000	3000.01	38.75	0.00	3000.01	38.78	0.00	3000.01	38.78	0.00
	4000	4000.40	38.81	−0.01	4000.01	38.83	0.00	4000.02	38.83	0.00
Jocky	1000	999.98	32.30	0.00	1000.01	32.40	0.00	1000.00	32.40	0.00
	2000	2000.03	35.55	0.00	2000.01	35.60	0.00	2000.00	35.61	0.00
	3000	3000.00	36.95	0.00	3000.04	36.97	0.00	3000.06	36.99	0.00
	4000	4000.00	37.68	0.00	3999.99	37.69	0.00	4000.02	37.71	0.00
Average			35.40	0.00		35.46	0.00		**35.51**	0.00

**Table 6:** PSNR Comparisons at different bit rates with other state-of-the-art rate control schemes.

Name of Video Sequence	Bit Rate	BA [44]	BAF [45]	RCA [37]	Proposed Method
FourPeople	384	36.30	36.81	37.07	**37.32**
	512	37.49	38.19	38.31	**38.38**
	850	39.76	39.98	40.03	**40.06**
	1200	40.52	40.69	40.89	**40.97**
BasketballDrillText	384	30.82	30.81	30.89	**30.99**
	512	31.87	31.86	31.91	**32.08**
	850	33.41	33.44	33.52	**33.60**
	1200	34.91	34.96	35.19	**35.32**

**Table 7:** Computational Complexity.

Intel Core i9-7960× CPU @ 2.80 GHz			
**Resolution**	**HM-16.10 (s/frame)**	**PS-GOP (s/frame)**	**Proposed Method (s/frame)**
WVGA	24.10	23.75	53.30
HD	45.18	44.92	97.79
Full HD	166.15	165.47	351.10
Average	78.48	78.04	167.40

## Data Availability

Not applicable.
